# Acute intermittent porphyria (AIP) in a patient with celiac disease

**DOI:** 10.1186/s42466-020-0049-6

**Published:** 2020-01-20

**Authors:** Sebastian Nunnemann, Christoph Uibel, Petra Budig, Mathias Mäurer

**Affiliations:** Department of Neurology, Klinikum Würzburg Mitte gGmbH, Standort Juliusspital, 97070 Würzburg, Germany

**Keywords:** Acute intermittent porphyria, Celiac disease, Polyneuropathy, Neurological rehabilitation

## Abstract

We present the case of an 18 year old Caucasian with known celiac disease, who suffered a severe first attack of acute intermittent porphyria (AIP) with neuropsychiatric symptoms, severe tetraparesis and respiratory insufficiency. Treatment with heme arginate and high-dose intravenous glucose and rigorous rehabilitation resulted in a slow but almost complete recovery of her motor symptoms. To our knowledge this is the first case of acute intermittent porphyria triggered by malnutrition in the context of celiac disease. It is remarkable that the patient showed a favourable outcome despite the severity of her initial symptoms. This case shows the importance of early and systematic symptomatic treatment in patients with severe neurologic manifestation of AIP.

## Introduction

Acute intermittent porphyria (AIP) is an autosomal dominant disorder, caused by an enzyme defect in the heme biosynthesis. Patients with AIP present with acute attacks with abdominal pain or autonomic dysfunctions. Some patients develop neuropsychiatric or neurological symptoms, [1–3].

Here we present a case of AIP in a woman with celiac disease, a combination which has not yet been described in the literature.

## Case report

An 18-year-old Caucasian woman was admitted to our medical department with abdominal pain, vomiting and loss of weight. Clinical and laboratory examinations showed celiac disease with positive gliadin-antibodies. Subsequent adjustment of her diet did not result in a relief of symptoms. Instead she developed neuropsychiatric symptoms and a progressive generalized motor weakness. She was admitted to the neurology department. Brain and spinal cord MRI, CSF analysis and nerve conduction studies did not reveal any pathological findings.

Considering the differential diagnosis of acute tetraparesis, we checked the urine for porphyrins and found high concentrations of delta-aminolevulinic-acid and porphobilinogen. Genetic testing revealed a heterozygous mutation of the exon 9 in the HMBS-gene (Mutation c.517C > T,p.Arg173Trp).

We therefore diagnosed an AIP with a severe motor neuropathy. Her medication was adjusted. A treatment with high dose intravenous glucose and heme arginate was started. Despite the treatment the patient deteriorated and needed mechanical ventilation and a tracheostomy. Two weeks after admission she was tetraplegic. Her clinical condition and nerve conduction studies revealed a prominent axonal loss.

We repeated the treatment with heme arginate and the patient gradually improved. She got daily physiotherapy, occupational therapy, speech therapy and neuropsychological support. After six weeks she was able to sit without aid. After three months she was able to stand with the support of two people. After 4 months she was able to start walking with a walker. By this time the tracheal cannula was removed.

A year after the incident she was able to walk with peroneal splints.

It became evident that her body weight was crucial for symptom control, so we maintained a percutaneous endoscopic gastrostomy to ensure a high-caloric diet.

## Discussion

We describe a young woman with a severe attack of an AIP with neuropsychiatric symptoms and an axonal motor neuropathy. These pronounced neurologic symptoms rarely occur in patients with AIP, [1–5].

The precise mechanism how AIP causes neurological deficits is poorly understood. Heme synthesis takes place in bone marrow and in hepatocytes. The first step of heme biosynthesis is determined by the enzyme delta-aminolevulenic-acid–synthase (ALA-S) by two different isoforms. The liver isoform ALA-S1 is crucial for the development of AIP. It is controlled by the concentration of heme, [1, 2]. Due to a genetic defect of hydroxymethylbilane synthase, less heme is produced and hepatocytes accumulate the heme precursors delta-aminolevulinic acid (ALA) and porphobilinogen (PBG) which are believed to be neurotoxic, [1]. Heme is also relevant for the energy supply of axons, [1, 3, 6].

In our case AIP occurred in a patient with preexisting celiac disease. A combination of celiac disease and acute intermittent porphyria has not been described before, only some rare findings of a combination of a celiac disease and a variegate porphyria are known, [7, 8]. We believe that the catabolic state of the patient caused by her celiac disease has triggered the first attack of AIP.

This case demonstrates that it might be useful to consider AIP when patients with celiac disease develop neurological or neuropsychiatric symptoms.

Since new medical treatments are about to come it is even more important to establish the diagnosis of AIP. Meanwhile there are promising studies with si-RNA that inhibit the first enzyme of the heme biosynthesis.

This case also demonstrates the value of intensive neurorehabilitation in severe AIP. Although our patient suffered from severe flaccid tetraparesis for months, she eventually had a good outcome. There are clinical studies, that demonstrate a favorable outcome in terms of recurrent attacks or medical complications, [4, 5]. However, information on the outcome of patients with severe neurological symptoms is rare. It has been described, that comprehensive rehabilitation leads to a favorable outcome, [9]. A small Finnish study showed, that even patients with severe attacks can make a full recovery or substantial improvement. Still, patients with severe attacks often need more than 6 months to recover. Not only the neurological symptoms predict the outcome of the patients but the combination of multiple symptoms.

The neurological symptoms in combination with multiple other symptoms predict the outcome of patient with AIP [10]. Intensive rehabilitation should be considered for all patients with severe attacks after the initial treatment.

## Data Availability

Data sharing not applicable to this article as no datasets were generated or analyzed during the current study.

## Abbreviations

AIP: Acute intermittent porphyria
ALA: Delta-aminolevulinic acid
ALA-S: Delta-aminolevulenic-acid–synthase
CSF: Cerebrospinal fluid
HMBS: Hydroxymethylbilane synthase
MRI: Magnetic resonance imaging
PBG: Porphobilinogen
